# The effect of continuous positive airway pressure therapy on atrial fibrillation in patients with obstructive sleep apnea

**DOI:** 10.3389/fmed.2025.1509776

**Published:** 2025-01-28

**Authors:** Jiancheng Hu, Siyuan Zuo, Jiahui Qian, Fangfang Cheng, Dengji Wang, Yanyan Deng, Dasheng Lu

**Affiliations:** ^1^Department of Cardiology, The Second Affiliated Hospital of Wannan Medical College, Wuhu, Anhui, China; ^2^Scientific Research Department, The Second Affiliated Hospital of Wannan Medical College, Wuhu, Anhui, China; ^3^Vascular Diseases Research Center of Wannan Medical College, Wuhu, China

**Keywords:** obstructive sleep apnea, continuous positive airway pressure, atrial fibrillation, chronic intermittent hypoxia, atrial remodeling

## Abstract

Obstructive sleep apnea (OSA) stands as an autonomous risk factor for a broad spectrum of cardiovascular diseases, particularly atrial fibrillation (AF), which is closely associated with heightened morbidity and mortality rates. The intricate pathophysiological pathways linking OSA to AF encompass chronic intermittent hypoxia, disruptions in the autonomic nervous system, inflammatory responses, and alterations in ion channel function. Continuous positive airway pressure (CPAP) therapy emerges as the frontline treatment for moderate to severe OSA, effectively alleviating symptomatic manifestations and potentially mitigating cardiovascular risks. However, the influence of CPAP on AF among OSA patients remains a subject of debate. Some investigations underscore its beneficial effects, including the reversal of atrial remodeling, enhanced atrial conduction, decreased AF incidence, and improved outcomes post-AF ablation in CPAP-treated individuals. Conversely, other studies reveal neutral or insignificant impacts. This review delves into the repercussions of CPAP therapy on AF in OSA patients, exploring potential explanations for the discrepancies observed across existing research endeavors. By consolidating current evidence and pinpointing areas ripe for further inquiry, this review aspires to inform clinical decision-making regarding the management of OSA-related AF.

## Introduction

Obstructive sleep apnea (OSA) represents a widespread sleep disorder across the globe (1), distinguished by recurrent episodes of upper airway obstruction during sleep, resulting in intermittent airflow reduction or cessation and disrupted breathing patterns (2). The majority of OSA patients exhibit structural narrowing in the upper airways, encompassing the nose and pharynx. These abnormalities may include nasal obstruction (stemming from rhinitis, deviated nasal septum, turbinate hypertrophy, polyps, or nasal tumors), tonsillar hypertrophy, redundant soft palate, elongated or thickened uvula, pharyngeal stenosis, pharyngeal tumors, tongue hypertrophy, tongue base retroflection, mandibular retrusion, temporomandibular joint dysfunction, and micrognathia. Such anatomical variations contribute to elevated airway resistance, obstruction, and collapse (3, 4).

Worldwide, it is estimated that approximately 1 billion adults are affected by OSA (defined by an Apnea Hypopnea Index, AHI ≥ 5 events/hour), with roughly 425 million adults necessitating treatment for moderate to severe OSA (AHI ≥ 15 events/hour) (5). China leads in the prevalence of OSA patients (176 million), followed by the United States (54 million), Brazil (52 million), and India (49 million) (5). The male-to-female ratio among OSA patients ranges from approximately 2:1 to 4:1, with a notable surge in prevalence observed among postmenopausal women (6).

Polysomnography serves as the definitive diagnostic tool for OSA, typically involving at least 7 h of sleep monitoring while recording electroencephalogram, electromyogram, oro-nasal airflow, chest and abdominal movements, oxygen saturation, and electrocardiogram (7). The AHI is a pivotal parameter for diagnosing OSA and assessing its severity, classifying OSA as mild, moderate, or severe based on AHI and the lowest nocturnal oxygen saturation (Table 1) (8).

**TABLE 1:** Classification of obstructive sleep apnea.

Severity	AHI (events/hour)	Lowest nocturnal SaO2 (%)
Mild	5∼15	85∼90
Moderate	>15∼30	80∼<85
Severe	>30	<80

Research has implicated OSA as an independent risk factor for various cardiovascular diseases (CVD) (9). Not only does OSA augment the risk of CVD, but it also exacerbates CVD outcomes (10). The adverse consequences of OSA are linked to an increased burden of arrhythmias (11), particularly influencing the onset, progression, treatment, and recurrence of atrial fibrillation (AF) (12–15). Through mechanisms such as autonomic nervous system dysfunction, chronic intermittent hypoxia (CIH), hypercapnia, alterations in intrathoracic pressure, inflammation, and ion channel changes, OSA may induce structural and electrical remodeling of the atrium. Consequently, OSA patients exhibit a higher incidence of AF, greater difficulty in achieving drug/electrical cardioversion, and an elevated risk of AF recurrence following restoration of sinus rhythm.

Continuous positive airway pressure (CPAP) constitutes the primary treatment modality for patients with moderate to severe OSA. CPAP effectively mitigates OSA symptoms, including daytime sleepiness, fatigue, headache, and memory impairment (16). A substantial proportion of OSA patients experience a reduced risk of CVD events and improved long-term CVD outcomes following CPAP therapy (17–19), although some studies have failed to demonstrate such benefits (20–23). The current literature on the impact of CPAP on OSA-related AF yields conflicting results, with some studies indicating positive effects, while others report neutral outcomes in clinical trials (24–26). These inconsistencies underscore the necessity for further investigation into the effects of CPAP on AF in OSA patients, which is crucial for informing clinical decisions regarding treatment and prognosis.

**FIGURE 1:**
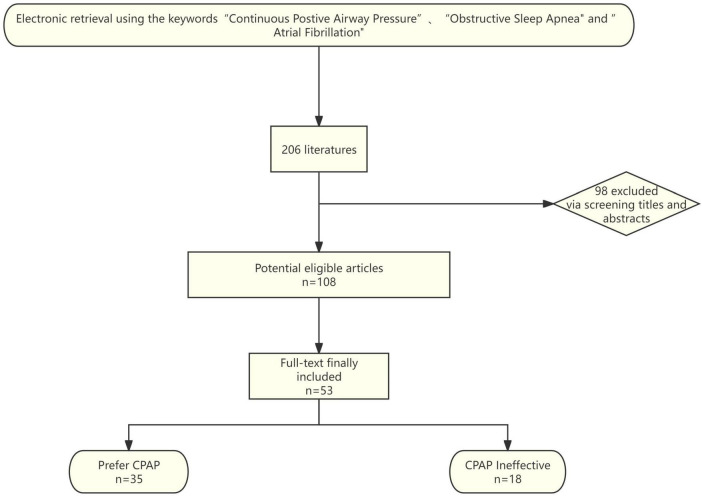
Flowchart of literature search and inclusion.

This review aims to summarize the impact of OSA on AF, delve into the potential pathophysiological mechanisms that link OSA to the occurrence, development, treatment, and prognosis of AF, and discuss the influence of CPAP therapy on AF, while analyzing potential reasons for the variability in study outcomes. We conducted electronic searches using the keywords ‘sleep apnea’, ‘atrial fibrillation’, and ‘continuous positive airway pressure’ up to July 2024, focusing on the PubMed database. By examining the titles and abstracts of the retrieved articles, we identified relevant studies, and those meeting the criteria were subjected to full-text review for further evaluation. Figure 1 shows the flow chart.

## The adverse effect of obstructive sleep apnea on atrial fibrillation

AF represents the most prevalent sustained cardiac arrhythmia worldwide, affecting an estimated 2–4% of the population, or over 33 million individuals (27). This condition is associated with heightened risks of stroke, heart failure, cognitive impairment, hospital admission, and mortality (28). Notably, studies indicate that 21–74% of AF patients also endure OSA, and the incidence of AF among those with OSA is 88% greater than in the general population, hinting at a potential linkage between these two disorders (29). Shared risk factors, including obesity, hypertension, congestive heart failure, and advanced age, may underlie this association (30).

Research has demonstrated that OSA can exacerbate the onset and progression of AF. Monahan et al. conducted a study involving 61 AF patients undergoing antiarrhythmic drug (AAD) therapy, all of whom underwent polysomnography. Their findings revealed a correlation between nocturnal paroxysmal AF and the presence of OSA (31). Furthermore, the study documented that OSA can diminish the efficacy of AAD, electrical cardioversion, and catheter ablation in managing AF (32). Additionally, persistent research has shown that AF patients with OSA exhibit a significantly elevated recurrence rate following CA procedures (33–36). Matiello et al. investigated 174 patients who underwent polysomnography and received circumferential pulmonary vein isolation (PVI), with subsequent 24- or 48-h dynamic electrocardiogram monitoring at specified intervals. Any instance of AF or atrial flutter was deemed a recurrence. In this study, the 1-year arrhythmia-free probability after a single ablation was 48.5% for mild OSA, 30.4% for moderate OSA, while both pre- and post-procedural left atrial diameter and severe OSA emerged as independent predictors of arrhythmia recurrence (34). Tang et al. conducted a clinical trial encompassing 178 patients with paroxysmal AF who underwent PVI. Using the Berlin Questionnaire, they stratified the OSA group into high-risk and low-risk subgroups. No statistically significant difference in recurrence rates was observed between the high-risk and low-risk subgroups (36). In another trial by Kanagala et al., among 39 OSA patients, 27 received no CPAP treatment or used CPAP incorrectly. These 27 patients exhibited an AF recurrence rate of 82% at 12 months, compared to a 42% recurrence rate in the treated OSA group (33).

### Changes in intrathoracic pressure

During inhalation, obstruction of the upper airways leads to an increase in intrathoracic pressure. This heightened pressure not only worsens airway collapse and obstruction but also exerts greater force on both the atrial and ventricular walls (37). The resultant increased tension on the atrial walls accelerates the chronic progression of atrial dilation, with this dilated atrium subsequently initiating the onset of AF (37). Atria that are acutely dilated are susceptible to inducing a variety of atrial arrhythmias (38). It should be noted that this is one hypothesis and that other mechanisms may also be involved.

### Autonomic nervous system dysregulation

In patients with OSA, rapid alterations in cardiac autonomic balance occur, varying with the different stages of acute apneic episodes. Typically, during apnea, there is an overactivation of the parasympathetic nervous system, manifesting as arrhythmias primarily characterized by bradycardia and conduction blocks. Conversely, during the resumption of breathing, there is a rebound excitation of the sympathetic nervous system, with heart rate normalizing or becoming tachycardic (32). Yu et al. reported that OSA notably elevates the inducibility of AF and augments left renal sympathetic nerve activity; however, these activities can be inhibited through low-level tragus vagal stimulation (15). Xiaokereti et al., utilizing a dog model, demonstrated that OSA significantly enhances neural remodeling in both the left stellate ganglion and left atrium. Excessive activity of the left stellate ganglion may expedite left atrial neural remodeling, thus augmenting AF inducibility (39). Linz et al., employing a pig model, indicated that negative tracheal pressure during obstructive events is a potent trigger for AF. Negative tracheal pressure primarily leads to a shortening of the atrial effective refractory period and increases vulnerability to AF by activating the vagus nerve (40). Furthermore, hyperactivity of the sympathetic nervous system can accelerate atrial fibrosis via activation of the renin-angiotensin-aldosterone system. Goette et al. found that, compared to sinus rhythm, the expression of angiotensin-converting enzyme during persistent AF is elevated threefold (41).

### Chronic intermittent hypoxia

OSA, resulting from either complete or incomplete ventilation restriction, leads to the development of CIH within the body. A multitude of studies have provided evidence that CIH plays a mediating role in atrial structural remodeling, thereby exerting detrimental effects on the inducibility of AF (42–48). In an OSA model established by Ramos et al., histological analysis revealed a 43% increase in the interstitial collagen fraction in the atria of OSA rats compared to the control group, while no such difference was observed in the ventricles (44). Linz also employed a rat model, subjecting the rats to intermittent negative airway pressure (12 times per hour) to simulate mild to moderate OSA, with rats not exposed to intermittent negative airway pressure serving as the control group (CTR). The study found that acute intermittent negative airway pressure increased susceptibility to atrial oxidative stress. In the chronic test series, although atrial oxidative stress did not accumulate, histological analysis of the atria revealed an increase in cardiomyocyte diameter, a decrease in connexin 43 (CX43) expression, and an increase in interstitial fibrosis formation (43).

Yang et al. conducted a study where 60 male Sprague-Dawley rats were randomly divided into four groups: control group, CSD (cardiac sympathetic denervation) group, CIH group, and CIH + CSD group. They analyzed cardiac structure using HE staining and echocardiography, detected CX43 and tyrosine hydroxylase using Western blot, immunohistochemistry, and immunofluorescence, and recorded blood pressure, blood gas, heart rate, etc. The study demonstrated that CIH induces atrial remodeling, increases AF inducibility, leads to excessive sympathetic innervation, and decreases CX43 expression (46). Four years later, Yang et al. further conducted *in vivo* experiments using OSA rats and *in vitro* experiments using the CIH H9c2 cell model to investigate the role and potential mechanisms of Cx43 in OSA-related AF. The study revealed that Cx43 overexpression inhibits the expression of calcium/calmodulin-dependent protein CaMKII.γ through the Cx43/CaMKIIγ/HIF-1 axis, ultimately reducing myocardial apoptosis and the incidence of AF (45). Bober et al. focused on examining the effects of CIH on atrial electrophysiology and arrhythmia susceptibility. The study found that CIH increases susceptibility to atrial arrhythmia induction, primarily due to parasympathetic activation. The enhanced susceptibility to AF is accompanied by increased electrophysiological responses of atrial muscle to carbachol and isoproterenol, reduced response to propranolol, and elevated atrial M2 receptor protein levels (42).

### Hypercapnia

As a consequence of recurrent upper airway obstruction, hypoxemia is frequently accompanied by hypercapnia, which further exacerbates the risk of AF. Stevenson et al. developed a sheep model of hypercapnia and observed a significant prolongation of the effective refractory period by 152%, alongside an increase in conduction time relative to baseline measurements. Upon resolution of hypercapnia, the ERP promptly reverted to baseline levels; however, the recovery of conduction time was notably delayed by an average of 117 ± 24 min. This disparity in the recovery rates between ERP and conduction time may underlie the heightened susceptibility to AF witnessed during the post-hypercapnia period (49).

### Inflammation

A growing body of evidence implicates a strong correlation between inflammation and AF. Elevated serum levels of inflammatory markers have been documented in AF patients, with expression also noted in cardiac tissue. Furthermore, anti-inflammatory drugs have demonstrated efficacy in AF animal models. Specifically, AF patients exhibit heightened circulating levels of inflammatory markers, including C-reactive protein, interleukin-6, interleukin-8, interleukin-10, and tumor necrosis factor-α. It is noteworthy that these marker levels are typically higher in patients with persistent AF than in those with paroxysmal AF (50). Inflammation plays a pivotal role in modulating cell signaling pathways pertinent to fibrosis, apoptosis, and hypertrophy, which can facilitate structural remodeling of the atrium and augment the propensity for AF induction (51, 52).

### Ion channel alterations

Cellular bioelectricity arises from the movement of charged ions through cell membranes, facilitated by ion channels. In individuals with AF, electrical remodeling takes place within the atrium, encompassing alterations in various ion channels (47, 53). Zhang et al. conducted a study where they randomly assigned 80 male Sprague-Dawley rats into two groups: a control group and a CIH group, with 40 rats in each. Utilizing RT-qPCR, Western blot, and immunohistochemistry techniques, they assessed the expression levels of ion channel subunits. Their findings revealed a significant reduction in the densities of INa, ICa-L, and Ito in the CIH group compared to the control (53).

### The effect of CPAP on OSA-related AF

Table 2 listed representative studies on the effects of CPAP on Patients with OSA and AF.

**TABLE 2:** Representative studies on the effects of CPAP on patients with OSA and atrial fibrillation.

References	Baseline Characteristics	Average nightly usage time of CPAP	Follow-up time	Conclusions	Study design
Fein et al. (68)	PVI(+)/OSA(+)/CPAP(+): [Mean age: 57 years, 77% male, BMI: 29] PVI(+)/OSA(-)/CPAP(-): [Mean age: 59 years, 72% male, BMI: 30] PVI(-)/OSA(+)/CPAP(+): [Mean age: 55 years, 73% male, BMI: 31] PVI(+)/OSA(-)/CPAP(-): [Mean age: 59 years, 72% male, BMI: 30]	4.2 h	Outpatient follow-up at 1, 3, 6, and 12 months	CPAP can improve AF-free survival rate (71.9% vs. 36.7%; *p* = 0.01) and AF-free survival rate after repeat ablation following PVI (65.6% vs. 33.3%; *p* = 0.02)	Prospective controlled study
Traaen et al. (26)	CPAP(+): [Mean age: 63 years, 39% male, BMI: 30] CPAP(-): [Mean age: 62 years, 43% male, BMI: 29]	4.4 h	follow-up from 2 to 5 months using implantable loop recorder	Treatment with CPAP did not result in a statistically significant reduction in AF burden	Randomized controlled trials
Zhou et al. (2)	PVI(+)/OSA(+)/CPAP(+): [Mean age: 60 years, 45% male, BMI: 30] PVI(+)/OSA(+)/CPAP(-): [Mean age: 62 years, 46% male, BMI: 30] PVI(+)/OSA(-)/CPAP(-): [Mean age: 61 years, 44% male, BMI: 29	5.2 h	12-month follow-up using ambulatory electrocardiogram	OSA patients who received CPAP treatment had a higher AF-free survival rate (70.3% vs 31.5%; *P* = 0.02). CPAP significantly improved the arrhythmia-free survival rate and reduced the recurrence of AF in AF patients with OSA after RFCA.	Prospective controlled study
Srivali et al. (72)	All OSA patients: [Mean age: 64 years, 75% male, BMI: 35] CPAP group: [Mean age: 64 years, 78% male, BMI: 36] Control group: [Mean age: 62 years, 69% male, BMI: 34]	More than 4 h	4.6-month follow-up	CPAP treatment had no effect on the time to recurrence of AF after AF intervention	Single-center, retrospective study
Hunt et al. (25)	CPAP group: [Mean age: 62 years, 28% male, BMI: 30] Non-CPAP group: [Mean age: 62 years, 43% male, BMI: 30]	More than 4 h	3-to-12-month follow-up	The AF burden was reduced after ablation in both groups, and there was no difference between the groups.	Randomized controlled trials
Caples et al. (24)	CPAP group: [Mean age: 64 years, 58% male, BMI: 36] Non-CPAP group: [Mean age: 65 years, 54% male, BMI: 36]	6 h and 11 min	12-month follow-up using electrocardiogram	No difference was found between patients receiving CPAP and those receiving standard care	Randomized controlled trials

PVI, pulmonary vein isolation; CPAP, continuous positive airway pressure; OSA, obstructive sleep apnea; BMI, body mass index.

#### Positive effects of CPAP on AF

##### Reversal of atrial remodeling

Macedo et al. enrolled patients who had recently been diagnosed with metabolic syndrome and moderate-to-severe OSA to undergo a 6-month treatment regimen with either CPAP or nasal dilators (serving as a placebo). During the follow-up period, a notable enlargement of the atrial diameter was observed in the placebo group, increasing from 39.5 to 40.5 mm (*p* = 0.003). Conversely, the CPAP group exhibited stable atrial dimensions, with measurements remaining at 40.0 mm both before and after treatment. This randomized placebo-controlled trial suggests that a 6-month course of CPAP therapy can effectively halt atrial remodeling in patients with concurrent metabolic syndrome and OSA (54).

Nalliah et al. conducted a study involving 24 patients with OSA-associated AF, assigning them to either CPAP treatment (*n* = 12) or no CPAP (*n* = 12). All participants underwent invasive electrophysiological assessments at baseline and following a minimum of 6 months, where parameters such as atrial voltage (mV), conduction velocity (m/s), atrial surface area < 0.5 mV (%), proportion of complex points (%), and atrial effective refractory period (ms) were recorded. The study revealed a significantly faster conduction velocity in the CPAP group, coupled with a markedly higher voltage and a lower proportion of complex points. The findings of this randomized trial indicate that CPAP therapy can reverse atrial remodeling in AF patients (55).

Müller et al. reported that following CPAP treatment, there was a significant reduction in the pulsed-wave tissue Doppler imaging interval and a substantial decrease in BNP levels, signifying reversal of atrial remodeling in OSA patients receiving CPAP (56). Vural et al. utilized two-dimensional speckle tracking echocardiography to assess left atrial function in OSA patients undergoing CPAP therapy. Their study, which included 43 patients (AHI > 20 events/hour) treated with CPAP for 24 weeks, found that abnormal left atrial volume and deformation in OSA patients could be normalized within 12 weeks of CPAP therapy, with gradual improvement in left atrial anatomical remodeling over 24 weeks (57).

Regarding improved atrial conduction, Russo et al. conducted a study comparing 50 patients with OSA to 50 obese controls matched by gender and age. The study highlighted the positive impact of CPAP therapy on atrial conduction time and its potential to reduce AF incidence in OSA patients. After 6 months of CPAP treatment, significant improvements were noted in interatrial, intra-left atrial, and right atrial electromechanical delay, as well as the maximum P-wave duration and dispersion (58). Similarly, Bayir et al. demonstrated significant improvements in interatrial, intra-left atrial, and right atrial electromechanical delay, as well as P-wave dispersion, after 6 months of CPAP treatment, suggesting that CPAP therapy promotes more uniform atrial conduction, thereby mitigating the risk of OSA-related AF (59). Maeno et al. measured the signal-averaged P-wave duration (SAPWD) in participants, including 62 patients with moderate-to-severe OSA who were reassessed after 1 month of CPAP treatment, and a control group of 18 patients with moderate-to-severe OSA who were not treated with CPAP. The results indicated no significant change in SAPWD in the control group after 1 month. However, SAPWD was significantly shortened in the CPAP-treated group, with a statistically significant difference in SAPWD change between the two groups. Additionally, the change in SAPWD in the CPAP-treated patients was negatively correlated with the nightly duration of CPAP use (60).

##### Reducing AF incidence

A prospective longitudinal investigation conducted by Varga and colleagues enrolled 93 individuals diagnosed with OSA, who underwent in-home sleep apnea assessments and ambulatory electrocardiogram monitoring both at the time of diagnosis and following 3 months of CPAP therapy. When compared to baseline measurements, the occurrence of AF after 3 months of CPAP treatment exhibited statistical significance (*P* = 0.03), and AF correlated with the longest apnea duration (*r* = 0.215; *P* = 0.04). Notably, the prevalence of AF among OSA patients declined after 3 months of CPAP therapy (61). Saito et al. conducted a retrospective cohort analysis examining periodic breathing (PB) rates in OSA patients and factors influencing PB occurrence or persistence. They reported a median PB rate of 0.32% among those receiving CPAP therapy. Regression analysis identified AF as the most significant factor contributing to PB, with a coefficient of 0.693 (95% CI; 0.536–0.851), suggesting that CPAP therapy decreased AF incidence (62).

##### Enhanced success and reduced relapse in AF ablation

**FIGURE 2:**
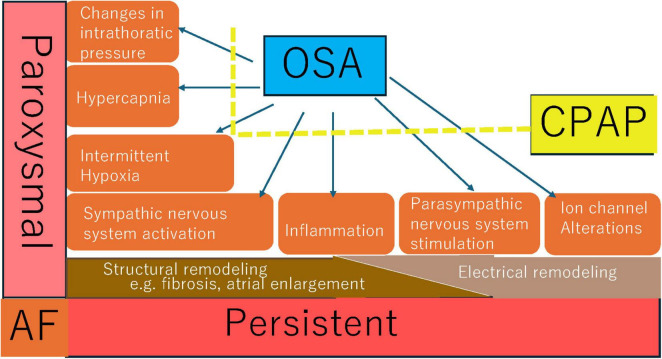
Possible mechanisms of CPAP in improving atrial remodeling and atrial fibrillation induced by OSA.

Several studies have demonstrated that initiating CPAP therapy prior to AF catheter ablation enhances ablation success rates (63, 64). Patel et al. performed a comparative study to evaluate whether CPAP therapy could augment the success rate of PVI. The study revealed that CPAP therapy improved PVI success, with patients not receiving CPAP therapy experiencing an eight-fold higher risk of procedural failure (64). Li et al. conducted a meta-analysis encompassing nine studies with a total of 14,812 patients. According to the random-effects model, CPAP therapy reduced the risk of AF relapse or progression by 63%. Compared to patients without OSA, those with OSA who did not use CPAP faced a significantly higher risk of AF recurrence or progression (65). Zhou et al. divided 122 OSA patients who had undergone radiofrequency catheter ablation (RFCA) into two groups, with 62 patients receiving CPAP therapy and 60 patients not. After 12 months of follow-up, the study found that OSA patients using CPAP therapy had a higher AF-free survival rate compared to those not using CPAP, indicating that CPAP therapy significantly improved arrhythmia-free survival and reduced AF recurrence in OSA patients after RFCA (66). Congrete et al. included seven observational studies with a total of 4,572 AF patients who had successful catheter ablation. Among AF patients with OSA post-ablation, CPAP use was significantly associated with a decreased risk of AF recurrence, with a pooled odds ratio (OR) of 0.28 (67). Fein et al. compared 62 OSA patients divided into a CPAP group (*n* = 32) and a non-CPAP group (*n* = 30) with a cohort of non-OSA patients after pulmonary vein isolation (PVI). The study found that CPAP therapy improved AF-free survival and AF-free survival after repeat ablation following PVI. The AF recurrence rate in the CPAP group was comparable to that of the non-OSA group (68). Naruse et al. analyzed data on CPAP use and AF recurrence in 82 patients, with another 34 OSA patients forming the non-CPAP group. Over a mean follow-up of 18.8 ± 10.3 months, 51 patients had AF recurrence post-ablation. Cox regression analysis showed that left atrial volume, concomitant OSA and CPAP therapy were associated with AF recurrence during follow-up. Adequate CPAP therapy in OSA patients was linked to a lower AF recurrence rate (69). Neilan et al. reported that during a 42-month follow-up, 245 patients experienced AF recurrence: the incidence was 51% in OSA patients, 30% in non-OSA patients, 68% in untreated OSA patients, and 35% in treated OSA patients. The study indicated that CPAP therapy was associated with a reduced risk of AF recurrence following PVI (35). In Abe et al.’s study, CPAP therapy significantly decreased the incidence of PAF, PVC, sinus bradycardia, and sinus arrest (70). The mechanisms by which CPAP improves atrial remodeling and AF caused by OSA are multifaceted. Figure 2 summarizes some mechanisms that may be relevant.

### Neutral effects of CPAP on AF

A retrospective analysis conducted by Ogbu et al. examined individuals diagnosed with unstable angina, acute myocardial infarction, acute decompensated heart failure, and AF with rapid ventricular response, who concurrently had OSA, utilizing data from the National Inpatient Sample spanning 2016 to 2019. The findings revealed that, among patients with OSA, the application of CPAP led to a significant increase in both the duration of hospital stay (with an adjusted mean difference of 1.49 days) and hospital expenses (adjusted mean difference of $1,168). The routine administration of CPAP during acute cardiovascular episodes may potentially attenuate or nullify the beneficial adaptive responses elicited by chronic, intermittent ischemic stress (71). In a randomized controlled trial, Traaen et al. enrolled 108 participants with paroxysmal AF and moderate-to-severe OSA to evaluate the differences in AF burden (defined as the percentage of time spent in AF) between those who received CPAP in addition to standard care and those who received only standard care. The results indicated that, over the final 3 months of CPAP intervention, the mean time spent in AF decreased from 5.6% at baseline to 4.1% in the CPAP group, and from 5.0 to 4.3% in the control group. Traaen et al. concluded that CPAP therapy did not yield a statistically significant reduction in AF burden among patients with paroxysmal AF and OSA (26). Hunt et al. conducted another randomized controlled trial, where 37 patients were randomly assigned to the CPAP treatment group and 46 to the standard treatment group. Within 3–12 months following PVI, 57% of patients in both groups experienced at least one AF occurrence. The AF burden decreased after ablation in both groups, with no significant difference between them. The study’s conclusion was that CPAP treatment did not further diminish the risk of AF recurrence post-ablation (25). Caples et al. carried out the inaugural randomized controlled trial to explore the effect of OSA treatment on AF recurrence after direct current cardioversion. No significant difference was observed between patients who received CPAP treatment and those who received usual care. AF was reported in 25% of patients in both the CPAP and control groups, with durations of 129.0 ± 166.5 days and 109.3 ± 73.2 days, respectively. At follow-up, there were no notable differences in the Epworth Sleepiness Scale or the Functional Outcomes of Sleep Questionnaire (24). Srivali et al. performed a single-center retrospective study at a tertiary referral center, which included 30,188 patients with obstructive and central sleep apnea. Following the intervention, no significant difference was found in the time to AF recurrence between CPAP adherent users and non-users. The study’s findings indicated that CPAP treatment did not influence the time to AF recurrence after intervention (72).

## Discussion

Existing research presents a divided view on the efficacy of CPAP in addressing OSA-related AF, with some studies indicating positive effects while others suggest neutrality. It should be noted that existing reports may not adequately assess whether CPAP therapy, with sufficient duration and efficacy, was actually administered to the truly appropriate patients with OSA. The divergent results can be attributed to several factors as follows:

### Different results on CPAP effectiveness

The existing evidence shows divergent results regarding the effectiveness of CPAP treatment effectiveness in preventing the onset or recurrence of AF. Many studies have explored how CPAP can improve atrial remodeling or some electrocardiophysiological surrogate endpoints in patients with OSA. Some clinical studies have demonstrated that CPAP can reduce the incidence of AF in OSA patients. However, there is a lack of evidence regarding whether CPAP can delay the progression of AF to a persistent state. Furthermore, the impact of CPAP on AF incidence may also depend on the characteristics of the population, as its effects in the general OSA population may differ from those in patients who have undergone AF cardioversion (i.e., recurrence rates).

Additionally, the differences between the mixed apnea group and the obstructive apnea group need to be taken into account. The mixed apnea group, which often exhibits a high degree of respiratory instability, may not be directly comparable to the group primarily exhibiting obstructive apnea. Moreover, CPAP therapy is often less effective in patients with mixed apnea. When comparing outcomes across these groups, improvements in sleep-disordered breathing or atrial remodeling after CPAP therapy should be considered. These points require further exploration through more research in the future.

### Sample inadequacy

Many clinical studies, despite having a control group, only make comparisons to baseline and not to the control group in their results section. Furthermore, due to the relatively small number of patients enrolled, the statistical power of these studies may be affected, leading to inconsistent research conclusions at present. Larger randomized controlled trials are needed in the future to clarify the effect of CPAP on patients with AF.

### Variability in study designs

There is considerable variation in the design of experiments across studies. For example, Fein et al.’s investigation enrolled a prospective cohort of consecutive OSA and symptomatic AF patients who were referred for AF ablation surgery from July 2007 to January 2010. Conversely, Srivali et al. examined all OSA patients who underwent polysomnography between January 1992 and December 2014 and subsequently received AF interventions (ablation or cardioversion). These retrospective analyses may be susceptible to selection bias.

### Adherence to CPAP therapy

The average nightly usage of CPAP significantly affects the prognosis of OSA patients and potentially influences the outcomes of OSA-related AF as well. Ensuring adequate therapy duration and monitoring adherence is crucial for ensuring that CPAP is effective.

### Disparity in follow-up durations

The follow-up periods for assessing AF recurrence vary widely among studies, spanning from 2 to 12 months. This inconsistency cannot exclude the possibility that longer or shorter follow-up intervals might alter the observed AF recurrence rates.

Based on the current literature, the following are the key areas that future research should prioritize:

### Developing additional animal models

Current research exploring the impact of CPAP on AF in OSA patients primarily consists of clinical studies, with limited use of animal models. To gain a deeper understanding of the mechanisms and effects of CPAP in this context, future studies should establish more animal models to investigate the impact of CPAP on OSA-related AF.

### Boosting CPAP adherence

Inadequate adherence to CPAP therapy poses a significant challenge to effective OSA management. The reasons for non-adherence are multifaceted, encompassing social, psychological, and medical factors (73). Strategies to improve CPAP adherence may include comprehensive communication with patients and their families, the utilization of Automatic Positive Airway Pressure devices, and early administration of sedative-hypnotic medications, among other approaches. More importantly, future research needs to focus on establishing precise patient selection criteria to more accurately evaluate the effectiveness of CPAP in preventing or mitigating the onset of AF

### Investigating innovative OSA therapies

Beyond CPAP, several alternative treatment options exist for OSA, including lifestyle modifications, oral appliances, neuromuscular electrical stimulation, and surgical interventions. OSA patients urgently require more novel therapeutic strategies (74). Recent studies have shown that the combined use of anticholinergic drugs and norepinephrine agonists, such as AD109 (atomoxetine + aroxybutynin), can improve OSA. The mechanism underlying this combination therapy likely involves stimulating the upper airway dilator muscles and enhancing genioglossus responsiveness, thereby reducing sleep apnea episodes (75, 76).
